# Iranian Clinical Nurses’ Readiness for Self-Directed Learning

**DOI:** 10.5539/gjhs.v8n1p157

**Published:** 2015-05-15

**Authors:** Morteza Malekian, Sharzad Ghiyasvandian, Mohammad Ali Cheraghi, Akbar Hassanzadeh

**Affiliations:** 1 School of Nursing and Midwifery, Tehran University of Medical Sciences, International Campus (TUMS-IC), Tehran, Iran; 2 School of Nursing and Midwifery, Tehran University of Medical Sciences, Tehran, Iran; 3 School of Health, Isfahan University of Medical Sciences, Isfahan, Iran

**Keywords:** self-directed learning, lifelong learning, clinical nurses

## Abstract

**Introduction:**

Clinical nurses are in need of being able to adapt to the ever-changing environment of clinical settings. The prerequisite for their successful adaptation is to be lifelong learners. An approach for making nurses lifelong learners is self-directed learning.

**Aims:**

This study was undertaken to evaluate a group of Iranian clinical nurses’ readiness for self-directed learning and its relationship with some of their personal characteristics.

**Methods:**

This cross-sectional descriptive study was conducted in 2014. A random sample of 314 nurses working in three hospitals affiliated to Isfahan Social Security Organization, Isfahan, Iran, was recruited to complete the Fisher’s Self-directed Learning Readiness Scale.

**Findings:**

In total, 279 nurses filled the scale completely. The mean of their readiness for self-directed learning was 162.50±14.11 (120–196). The correlation of self-directed learning readiness with age, gender, marital status, and university degree was not statistically significant.

**Conclusion:**

Most nurses had great readiness for self-directed learning. Accordingly, nursing policy-makers need to develop strategies for promoting their self-directed learning. Moreover, innovative teaching methods such as problem solving and problem-based learning should be employed to prepare nurses for effectively managing the complexities of their ever-changing work environment.

## 1. Introduction

Rapid changes and steady advances in science and technology are among the main characteristics of clinical settings and hence, nurses need to be able to adapt to such ever-changing environments (Yuan, Williams, Fang, & Pang, 2012). Nurses’ success in adapting to unstable and ever-changing environments of clinical settings necessitates becoming lifelong learners and accepting great responsibility towards learning (Sharples & Moseley, 2010). An approach for making nurses lifelong learners is self-directed learning (Li, Deny, & Chen, 2009).

Self-directed learning (SDL) is a process in which individuals assume the responsibility of identifying their learning needs, setting goals, locating sources, developing and implementing proper strategies, and evaluating the outcomes of learning both individually and collectively (Knowles, Holton, & Swanson, 2008). The advantages of SDL include, but not limited to, gaining more autonomy in learning, having higher motivation for it, acquiring lifelong learning skills, and developing greater self-control, self-confidence, and self-management (O’Shea, 2003). Everybody can turn into self-directed learner; however, the level of their SDL ability depends on factors such as motivation for learning, self-confidence, conscience, experience, and intelligence (Cazan & Schiopca, 2014), which are collectively called readiness for SDL. According to Wiley (1983), SDL readiness is defined as “the degree the individual possesses the attitudes, abilities and personality characteristics necessary for self-directed learning”. Promoting nurses’ SDL readiness enhances their self-confidence, learning capacity, accountability, and independent learning ability (Bastable, 2013).

Currently, nursing is undergoing the transition to professionalization and hence, nurses need to competently perform their professional roles in such an evolving system (Hoseinpour & Heshmati Nabavi, 2012). Continuing education is among the main strategies which are used for enhancing nurses’ professional skills and helping them appropriately react to rapid changes in healthcare systems (Tomey, 2008). However, continuing education suffers from obvious shortcomings such as failure to involve nurses in the process of active learning (Vaezi, Vanaki, & Ahmadi 2013). Continuing education programs rarely motivate teacher-learner interactions and hence, relieve nurses of active learning responsibilities (James & Francis, 2011). According to Hosseini and Assareh (2011), an effective strategy for promoting nurses’ successful lifelong learning is SDL.

Before incorporating SDL techniques into formal nursing education, it is necessary to assess nurses’ readiness for it, i.e. their readiness for using creativity, problem solving, and assuming responsibilities (Smit, 1996). Identifying nurses’ SDL readiness would pave the way for revising teaching strategies and also for developing continuing education programs which effectively fulfills nurses’ educational needs.

Previous studies have reported that nursing students have varying levels of SDL readiness. For instance, Yuan et al. (2012) assessed 526 Chinese nursing students’ SDL readiness by using the Fisher’s Self-Directed Learning Readiness Scale (Fisher’s SDLRS). About 62.3% of their participants had great readiness for SDL. The mean of students’ SDL was 154.75±15.08. They finally concluded that students have the necessary requirements for SDL and hence, it should be incorporated into nursing curriculum. Klunklin et al. (2010) also employed the Guglielmino’s Self-Directed Learning Readiness Scale (Guglielmino’s SDLRS) to evaluate 272 Thai nursing students’ readiness for SDL and reported the same findings. In another study, Cadorin et al. (2012) evaluated registered nurses, radiology technicians, as well as nursing and radiology students’ readiness for SDL in Italy. They used the Williamson’s Self-Directed Learning Readiness Scale (Williamson’s SDLRS). On this scale, scores 221–300 reflect great readiness for SDL. They found that nurses, radiology technicians, and nursing and radiology students’ SDL readiness scores were respectively 229.1, 219.6, 212.3, and 222.4. Finally, they concluded that given the complexities and instabilities of clinical settings and patient care, SDL is a necessary skill for healthcare professionals and recommended implementing strategies for supporting, encouraging, and facilitating healthcare professionals’ SDL.

In our country, Iran, few studies dealt with assessing nursing students’ SDL readiness. For instance, Safavi et al. (2010) evaluated 178 Iranian nursing students’ readiness for SDL as well as their learning styles by employing the Fisher’s SDLRS and the Kolb’s Learning Styles Questionnaire, respectively. They reported that only 34.3% of their participants indicated great readiness for SDL and the remaining 65.7% were poorly or moderately ready for it. Karshki et al. (2013) also reported that Iranian nursing students’ SDL readiness mean score was 148.20, indicating optimum readiness for SDL.

Our literature review showed that previous studies mainly aimed at investigating nursing students’ SDL readiness and hence, clinical nurses’ readiness for it has been taken for granted. Consequently, this study was conducted to bridge this gap.

The aim of the study was to evaluate clinical nurses’ readiness for SDL and its relationship with some of their personal characteristics.

## 2. Methods

### 2.1 Setting, Sample and Sampling

This cross-sectional descriptive study was conducted in 2014. Study population consisted of all nurses who worked in hospitals affiliated to Isfahan Social Security Organization, Isfahan, Iran. Three hospitals were affiliated to this organization at the time of the study. The inclusion criterion was having a university degree in nursing. Nurses who participated in SDL or problem-solving workshops were excluded. We calculated the study sample size by using the findings of a study conducted by Naeimi, Bigdeli, and Soltani Arabshahi (2012) and the following formula,


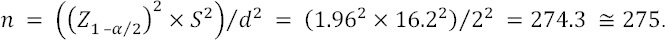


Consequently, with a confidence interval of 0.95, the sample size was determined to be equal to 275. However, by considering a probable attrition rate of 10%, we recruited 314 nurses to the study. Study participants were recruited by using the stratified random sampling technique. Accordingly, a quota was allocated to each hospital by considering its total number of practicing nurses and then, nurses were recruited proportionately.

### 2.2 Instrument and Data Collection

The study data collection instrument consisted of a demographic questionnaire and the Fisher’s SDLRS (2001). The demographic questionnaire was developed based on a literature review and contained items on participants’ age, gender, marital status, work experience, as well as their diploma and academic grade point averages (GPA). The Fisher’s SDLRS is a modified version of the Guglielmino’s SDLRS. The Guglielmino’s SDLRS (1978) is a self-report 58-item Likert-type scale. Fisher, King, and Tague (2001) evaluated the psychometric properties of the Guglielmino’s scale and reduced the number of its items to 42 and then to 40 (Smedley, 2007).

The 40-item Fisher’s SDLRS consists of three domains including self-control (fifteen items), desire for learning (twelve items), and self-management (thirteen items) (Fisher & King, 2010). The items of the Fisher’s SDLRS are scored on a five-point Likert scale from 5 (Completely agree) to 1 (Completely disagree). The self-control domain reflects that self-directed learners are completely independent individuals who can analyze, plan, implement, and evaluate their learning. The minimum and the maximum possible scores of this domain are 15 and 75, respectively. Self-management domain denotes that self-directed learners are able to identify their own educational needs, set learning goals, manage their time and energy for learning, and provide constructive feedbacks. The minimum and the maximum possible scores of the self-management domain are 13 and 65, respectively. Finally, the desire for learning implies that learners are strongly motivated for knowledge acquisition. The minimum and the maximum possible scores of this domain are 12 to 60 (Yousefy & Gordanshekan, 2011). Consequently, the total score of the Fisher’s SDLRS would range from 40 to 200.

The Cronbach’s alpha values for the self-control, desire for learning, and self-management domains and the whole Fisher’s SDLRS were respectively equal to 0.80, 0.85, 0.87, and 0.83, indicating the high reliability of the scale. Moreover, Fisher and King (2010) assessed and confirmed the construct validity of the scale by using the confirmatory factor analysis technique.

Nadi and Sajjadian (2006) translated the Fisher’s SDLRS into Persian. Initially, they asked an English-Persian translator, an educational expert, and a psychometric specialist to translate the scale into Persian. Then, they generated a unified Persian version of the scale and invited several students and several learning specialists to evaluate its face and content validity. After that, they asked an English language expert to back-translate the Persian version of the scale into English. The original and the generated English versions of the scale were identical, confirming the soundness of translations. Finally, Nadi and Sajjadian (2006) recruited 335 medical and dentistry students from two private universities located in Isfahan, Iran and asked them to complete the Persian Fisher’s SDLRS. They Used the LISREL software for data analysis and reported a Cronbach’s alpha, a Spearman-Brown coefficient, a Guttman’s coefficient, and a test-retest reliability coefficient of respectively 0.913, 0.899, 0.898, and 0.861 for the scale. Moreover, they found that all three domains of the scale had an acceptable internal consistency. We also invited fifteen nurses meeting the inclusion criteria to complete the scale. Then, the Cronbach’s alpha of the scale was calculated which was equal to 0.86.

We referred to the study setting and administered the study instrument to eligible nurses. Primarily, nurses were adequately informed about the instrument and how to complete it. Then, they were asked to complete the study instrument in fifteen minutes. Data collection was performed in May–July 2014.

### 2.3 Data Analysis

The SPSS v. 21.0 software was used for managing and analyzing the data. Study variables were described by emplying the measures of descriptive statistics such as frequency, percentage, mean, and stndard deviation. The independent-samples t test was used for comparing gender groups in terms of SDLRS scores. Moreover, the Pearson and the Spearman correlation tests were used for examining the relationship of SDLRS score with demographic variables such as age, educational and marital status, work experience, as well as the diploma and academic GPA.

### 2.4 Ethical Consideration

Written permission was obtained from the Isfahan Social Security Organization. Study participants were informed about the aim of the study and were ensured that participation in the study was voluntary. Study data were collected, analyzed, and reported anonymously. Written consent was obtained from the participants. Questionnaires were anonymous.

## 3. Results

In total, 314 questionaires were distributed, out of which, 297 were returned completely filled (i.e. a response rate of 0.94). Table 1 shows study participants’ personal characteristics. The means of participants’ ages and work experience were 28.37±3.56 and 11.30±6.20 years, respectively. Most of the participants were female (72.72%) and married (73.50%) and held bachelor’s degree (91.60%). Moreover, the means of participants’ diploma and academic GPA were respectively 16.73±1.40 and 16.54±1.02.

**Table 1:** Study participants’ personal characteristics (n=297).

Demographic Variables	n(%) Mean±SD
Age (years)	28.37±3.56
Gender:	
Male	81(27.27%)
Female	216(72.72%)
Marital Status:	
Single	79(26.50%)
Married	219(73.50%)
Academic Level:	
BSN	273(91.60%)
MSc	23(8.10%)
PhD	1(.30%)
Record of Service	11.36±6.20
Academic GPA	16.54±1.02
Diploma GPA	16.73±1.40

GPA= Grade Point Average, SD= standard deviation.

The mean of our participants’ SDLRS scores was 162.50±14.11. Table 2 shows the mean scores of SDLRS and its domains. The results of the independent-samples t test revealed that there were no significant differences between male and female as well as between single and married participants regarding the mean scores of SDLRS and its domains (P value > 0.05; Table 3). Moreover, the Spearman test showed that there was a week correlation between participants’ university degree and their desire for learning (r = 0.137 and P vvalue = 0.019).

**Table 2:** Study participants’ SDLRS (n=297)

	Subscale1: Self-management	Subscale2: Desire for Learning	Subscale3: Self-control	Overall score
Mean(SD)	47.78 (4.89)	53.41 (4.96)	61.31 (6.27)	162.50 (14.11)
Minimum	33	39	45	120
Maximum	59	65	75	196

**Table 3:** Correlation of participants’ characteristics with the mean scores of SDLRS and its domains

	Subscale1: Self-management	Subscale 2: Desire for Learning	Subscale 3: Self-control	Overall score
Gender:				
Male	48.29±5.10	53.57±5.22	61.71±6.41	163.58±14.73
Female	47.62±4.79	53.38±4.85	61.20±6.22	162.19±13.84
t-test	p=.287	p=.770	p=.529	p=.452
	p=.287	p=.770	p=.529	p=.452
Marital Status:				
Single	48.00±4.96	53.07±4.83	61.40±6.45	162.48±14.54
Married	47.82±4.93	53.61±4.92	61.51±6.25	162.94±13.99
t-test	T=.267	T=.786	T=.126	T=.235
	p=.790	p=.433	p=.900	p=.814
Age	r=-.017	r=-.018	r=-.065	r=.048
	p=.774	p=.758	p=.266	p=.416
Academic Level	r=.022	r=.137	r=.054	r=.070
	p=.704	p=.019	p=.361	p=.236
Record of Service	r=.139	r=.o41	r=.038	r=.080
	p=.017	p=.484	p=.512	p=.172
Academic GPA	r=.174	r=.110	r=.109	r=.147
	p=.030	p=.060	p=.062	p=.011
Diploma GPA	r=.099	r=.052	r=.012	r=.058
	p=.087	p=.375	p=.834	p=.319

In addition, the results of the Pearson test revealed that only the correlation between the participants’ work experience and their self-management skills as well as the correlation of their academic GPA with the mean scores of SDLRS and its self-management domain were statistically significant (P value < 0.05; Table 3).

## 4. Discussion

This study aimed atevaluating clinical nurses’ readiness for SDL and its relationship with some of their personal characteristics. The mean scores of SDLRS and its self-management, desire for learning, and self-control domains were respectively, 47.78±4.89, 53.41±4.96, 61.31±6.27, and 162.5±14.11. According to Fisher et al. (2001), scores higher than 150 show great SDL readiness while scores less than 150 reflect low SDL readiness. Accordingly, our participating nurses had high readiness for SDL. Yuan et al. (2012), El-Gilany and Abusaad (2013), and Jafari-Sani, Mohammadzadeh Ghaser, Garavand, and Hosseni (2012), also reported an SDLRS score of higher than 150 (154.72±15.08, 159.60±13.80, and 176.12±27.32, respectively). Safavi et al. (2010) also found that most of the nursing student participating in their study had great SDL readiness.

Our findings revealed that nurses had the necessary attitude, ability, and personality characteristics for SDL. Self-directed nurses are active, accountable, and motivated learners (Wang & Liu, 2008). Nonetheless, continuing education and in-service training programs neither have fulfilled Iranian nurses’ educational needs nor have improved their clinical performance and the quality of nursing care (Ebadi et al., 2012). The reason is that these programs do not actively involve nurses, i.e. the learners, in teaching-learning process. In other words, there is no active interaction between teachers and learners in these programs and hence, learners cannot share their experience (Hoseinpour & Heshmati Nabavi, 2012). Accordingly, teaching strategies that are more learner-centered should be employed to overcome such a shortcoming in continuing education programs. In learner-centered approaches to teaching, teachers have the role of a facilitator and learners assume the responsibilities of goal setting, learning, and outcome evaluation (Collins, 2004). Moreover, SDL workshops can be held for familiarizing nurses with SDL.

Study findings also revealed that SDL readiness was not significantly correlated with nurses’ age and gender. Chen, Wang, and Lin (2006) and Roberson and Merriam (2005) also reported the same finding. However, McCollin (2000), Nadi and Sajjadian (2011), and Nikitenko (2009) found that age and gender significantly affect SDL readiness. These conflicting findings can be attributed to the fact that Chen et al. (2006), Roberson and Merriam (2005), and we recruited a sample of adult learners while McCollin (2000), Nadi and Sajjadian (2011), and Nikitenko (2009) conducted their studies on younger students. Probably, adult learners have a firmer knowledge base and hence, their SDL readiness is not affected by age and gender. Naeimi et al. (2012) also noted that age cannot significantly contribute to adult learners’ self-directedness because they have already learned to adopt different learning styles for promoting their learning. Moreover, university-based educations are still somehow teacher-centered while post-university learning is largely self-directed.

On the other hand, the similarity between male and female nurses regarding SDL readiness can be due to the fact that the workplace environment and job specification were similar for all of our participants. We also found no significant relationship between our participants’ marital status and SDL readiness. This finding is in agreement with the findings reported by Derric, Rovai, Podon, Confessore, and Carr (2007) and El-Gilany and Abusaad (2013).

Primarily, it may seem that unmarried individuals have more free time for learning; however, it should be taken into account that self-directed learners have great self- and time-management skills (Kirwan, Lounsbury, & Gibson, 2014).

Our findings also showed that except for the desire for learning domain, our participants’ university degree was not significantly correlated with the mean scores of SDLRS and its self-control and the self-management domains. This finding is consistent with the findings of previous studies (Chen, 2006; Abu-Moghli, 2005; Safavi, 2010). This finding can be attributed to individuals’ higher aspirations for independence and self-directedness after the age of puberty. The significant correlation of the desire for learning domain with university degree is probably due to the fact that this domain reflects nurses’ motivation for learning while such a motivation is in turn significantly correlated with nurses’ desire for graduate and post-graduate educations (Winne & Nesbit, 2010).

We also found that while nurses’ SDL readiness was significantly correlated with their academic GPA, it had no significant correlation with their diploma average. Nadi, Yousefy, and Changiz (2011) also reported the same finding. However, Lounsbury et al. (2009) conducted a study on 398 guidance school, 568 high school, and 1159 university students and reported that students’ SDL readiness was not significantly correlated with their GPA.

This finding is probably due to the fact that in our country, Iran, secondary education is more teacher-centered than tertiary education (Mohammadi & Araghi, 2013). Finally, our findings revealed that there was no significant relationship between nurses’ readiness for SDL and their work experience. Shimpei, Kadedag, Hattori and Saito (2010) found that nurses who had longer work experience acquired lower SDL readiness scores.

### 4.1 Limitations

One of the study limitations was that the study sample consisted only of nurses who were affiliated to the Isfahan Social Security Organization. The second limitation was the use of a self-report measure. It is possible that a ceiling effect was present since all items were positively scored. Due to the multidimensional aspects of self-directed learning, both qualitative and quantitative approaches should be utilized to assess clinical nurses readiness for being self-directed learners (Yuan et al., 2012).

## 5. Conclusion

Study findings suggest that the participating nurses had high SDL readiness. In other words, nurses had self-control and self-management skills and thereby, can pave the way for their own learning.

Accordingly, nursing policy makers need to develop strategies for promoting their SDL. Moreover, continuing education programs need to be modified and revised for actively involving self-directed nurses in their learning and also for empowering them for lifelong learning. Finally, innovative teaching methods such as problem solving and problem-based learning should be employed to prepare nurses for effectively managing the complexities of their ever-changing work environment.
